# Robust vision transformer adaptation for UAV imagery

**DOI:** 10.3389/frai.2026.1867313

**Published:** 2026-09-03

**Authors:** Jagan Murugesan, P. Helen Vijitha, L. Jai Vinita, A. K. Parvathy, P. N. Jeipratha

**Affiliations:** School of Computer Science and Engineering, Vellore Institute of Technology, Chennai, India

**Keywords:** LayerNorm adaptation, post-earthquake assessment, test-time adaptation, UAV imagery, vision transformers, visual drift

## Abstract

Vision Transformers (ViTs) perform well on clean aerial imagery but degrade sharply when deployed in post-earthquake UAV operations, where motion blur, dust haze, illumination variation, and sensor noise combine to produce what we term *post-earthquake visual drift*, a structured distributional shift that can render an otherwise capable model dangerously unreliable in the field. Retraining or conventional domain adaptation is not a realistic option under the latency, compute, and annotation constraints of active disaster response. This work presents a comprehensive empirical evaluation of lightweight label-free test-time adaptation (TTA) methods for improving the robustness of ViTs under such deployment conditions, systematically comparing consistency-based self-supervision (MEMO) and entropy minimization (TENT) across datasets with varying classification complexity. Consistent with existing lightweight TTA approaches, only the LayerNorm affine parameters are adapted during inference, while the backbone and attention weights remain frozen. On the UAV-TEBDE post-earthquake dataset, accuracy rises from 73.47% under severe drift (severity 0.3) to 89.18% using MEMO, corresponding to a recovery of 15.71 percentage points over the drifted baseline, without using any ground-truth labels or performing any retraining. On UAV-TEBDE, MEMO also achieved higher recovery than the SAR baseline evaluated in this study. Additional evaluation on AID (30-class aerial scene classification) and UC Merced (21-class land-use classification) shows consistent drift-induced degradation and adaptation-driven recovery across datasets of varying complexity. The cross-dataset evaluation further reveals that the relative effectiveness of lightweight TTA methods depends on the classification-space dimensionality. TENT-based entropy minimization outperforms consistency-based MEMO when the class count is large, while MEMO is the stronger choice in low-class settings. This interaction between method design and classification-space dimensionality has direct consequences for choosing TTA strategies in operational disaster assessment pipelines. Feature-space PCA analysis confirms that LayerNorm-only updates geometrically recalibrate internal representations, rather than simply correcting the model's output logits.

## 1 Introduction

Unmanned aerial vehicles are now among the primary devices used following the occurrence of large-scale earthquakes. Scaling these decentralized UAV fleets for continuous disaster response also introduces critical systems-level requirements, such as establishing secure data task distribution frameworks via blockchain (Dong et al., 2025) and efficient energy sharing among distributed edge nodes (An et al., 2023). If the roads are too difficult to pass through, and there is no way for personnel to approach the region where an earthquake took place, UAVs supply images from above and thus allow for assessing the situation rapidly (Mowla, 2025). These are not passive reconnaissance roles; decisions made from UAV imagery directly shape where limited rescue capacity is directed, which means classification errors can carry serious humanitarian consequences.

Vision Transformers (ViTs) have proven their effectiveness in many applications within remote sensing, such as segmentation of urban scenes, detection of buildings' footprints on a large scale, and classification of land covers (Yi et al., 2023; Butt et al., 2024; Gibril et al., 2024). The key difference from the convolutional network is that ViTs utilize global self-attention, which considers the long-range spatial dependencies and not just the local receptive fields. In aerial imagery, where discriminative cues for damage categories, partial wall collapse, irregular roof geometry, debris scatter, can be distributed over relatively large image regions, this representational scope matters. Combine it with the availability of large-scale pre-trained weights and fine-tuning is practical even with modest domain-specific datasets, making ViTs a natural fit for GeoAI applications.

The difficulty is that ViTs trained on clean, well-curated imagery behave quite differently once they are actually in the field. Post-earthquake UAV operations consistently produce imagery that bears little resemblance to standard training data: platform vibration and wind gusts introduce motion blur; dust kicked up by building collapse, combined with smoke from secondary fires, creates layered haze that degrades contrast; rapidly shifting sunlight and shadow produce illumination gradients across a single image; and hardware stress elevates sensor noise beyond its nominal specifications. None of these degradations typically appears in training sets, and they rarely occur in isolation, it is their simultaneous combination that creates the distributional break we call *post-earthquake visual drift*. Formally, we define this drift as a compound, dynamic distribution shift where atmospheric (haze, illumination) and sensor/platform (noise, blur) corruptions exhibit spatial heterogeneity and temporal coherence across a flight path, geometrically distorting standard feature representations. This shift does not merely add noise to the model's predictions; it geometrically distorts the token representations produced by the transformer encoder, such that the decision boundaries learned during training no longer align with the shifted feature distributions. Our experiments confirm that a model achieving near-perfect accuracy on a clean held-out test set can lose over 20 percentage points under severe drift. The practical response to this problem, collecting labeled target-domain images and retraining, is simply unavailable in the middle of a disaster response operation, where time pressure, limited connectivity, and the complete absence of ground-truth annotations make any offline adaptation procedure infeasible (Zheng et al., 2024).

Test-Time Adaptation (TTA) offers a fundamentally different approach: rather than adapting before deployment, the model adapts continuously during deployment, using only the incoming unlabeled test stream and self-supervised signals derived from it (Wang et al., 2021; Liang et al., 2025; Zeng et al., 2024; Karimi and Dibeklioglu, 2026). The absence of any label requirement or stored target-domain data makes TTA inherently compatible with the operational constraints of disaster UAV systems, where there is no time to annotate examples and no infrastructure to transmit them for retraining. Recent lightweight TTA methods have demonstrated that adapting only the affine parameters of LayerNorm layers provides an effective balance between computational efficiency and adaptation capability while avoiding updates to the full network parameters (Zhao et al., 2024). Despite the growing TTA literature, however, existing methods have been evaluated almost exclusively on standard computer vision benchmarks. Their behavior under post-earthquake UAV imagery, particularly across datasets with substantially different classification complexities, remains insufficiently explored.

**Figure 1:**
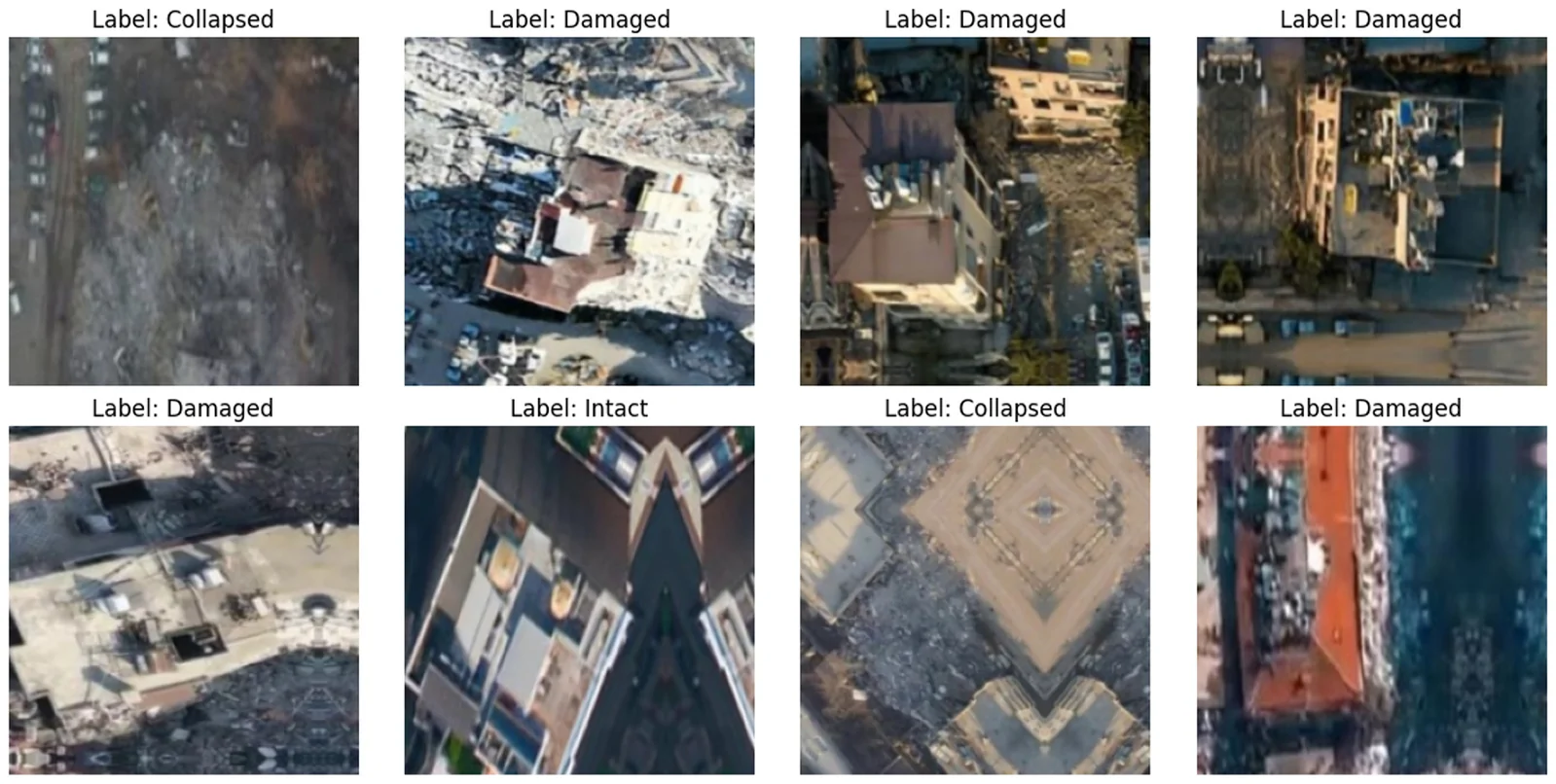
UAV-TEBDE: sample imagery from the three building damage categories, Collapsed, Damaged, and Intact. This is the primary post-earthquake benchmark used for adaptation experiments. Images were captured following the 2023 Turkey earthquakes. Images were sourced from (Ref: Mowla, Md. Najmul (2025), “UAVs-based Turkey Earthquake Building Damage Estimation Dataset (UAVs-TEBDE)”, Mendeley Data, V1, doi: 10.17632/5m349hfvkb.1). The files associated with this dataset are licensed under a Creative Commons Attribution 4.0 International licence.

This paper directly addresses those gaps. Rather than proposing a new LayerNorm adaptation strategy, this work presents a comprehensive empirical evaluation of lightweight label-free TTA methods for Vision Transformers operating under post-earthquake visual drift. Following established lightweight adaptation approaches, only the LayerNorm affine parameters are updated during inference, while the backbone and attention weights remain frozen. The study systematically compares consistency-based MEMO and entropy-minimization-based TENT under a common evaluation protocol and further investigates SAR on the primary UAV-TEBDE benchmark. On UAV-TEBDE, MEMO achieves a 15.7 percentage-point recovery under severe visual drift (severity 0.3). The recovery is quantified from the difference between the drifted baseline and the adapted accuracy, as detailed in the experimental results. Applying the same evaluation protocol across UAV-TEBDE (3 classes), AID (30 classes), and UC Merced (21 classes) reveals a clear and repeatable method-complexity interaction that has not, to our knowledge, been previously reported: consistency-based MEMO is the better strategy when the class space is small, while entropy minimization (TENT) is substantially superior when the class space is larger. We also provide PCA-based geometric evidence that LayerNorm updates act at the representation level, not just at the output logit level. The three evaluation datasets are illustrated in Figure 1.

## 2 Related work

### 2.1 UAV-based disaster assessment

The analysis of UAV imagery collected after an earthquake using automated techniques has become an area of active investigation lately, mainly due to the increased quality of drone sensors. For example, multimodal approaches, such as ReliefNet (Rahman et al., 2026), integrate visual input together with context to better classify the severity in real time. Moreover, it has been reported that integrating synthetic aperture radar (SAR) coherence with optical imagery is able to increase accuracy for damaged buildings identification when only optical imagery cannot provide enough information about them (Yang et al., 2024). Unsupervised domain adaptation algorithms can be used as well in order to minimize dependence on annotated samples in the target domain (Zheng et al., 2024).

That said, most of these systems are designed with the implicit assumption that imaging conditions are at least moderately stable, or that representative target-domain samples can be accessed at some point during system preparation. The particular challenge of heterogeneous, simultaneous corruptions, where motion blur, haze, noise, and illumination variation all occur in the same image at the same time, is largely unaddressed. The distinction matters: a model or adaptation method that handles each degradation in isolation may fail in a qualitatively different way when multiple corruptions co-occur, because their interactions can produce feature distortions that are not simply the sum of their parts.

### 2.2 Transformers in remote sensing

Within remote sensing specifically, ViTs and their hybrid variants have become a widely adopted choice across a broad range of perception tasks. UAVformer demonstrated competitive performance on urban scene segmentation from drone imagery (Yi et al., 2023); graph-infused hybrid transformers have been shown to improve land cover classification in GeoAI applications (Butt et al., 2024); and transformer-based semantic segmentation pipelines have been used for large-scale building footprint extraction from satellite imagery (Gibril et al., 2024). Semi-supervised and multimodal variants extend these capabilities to scenarios where labeled data is scarce (Li et al., 2026), which is frequently the case in disaster response contexts.

A recurring and relevant observation across robustness studies is that ViTs tend to handle mild distribution shift better than CNNs, likely because global attention distributes representational load across the full token sequence rather than relying on local filters that are sensitive to local texture changes. However, under severe or structured corruptions, ViTs are not robust (Liu et al., 2024); the shift in input statistics propagates through the encoder and disrupts the layer activations in ways that classification heads trained on clean data cannot accommodate.

The stability of transformer architectures is closely linked to the behavior of Layer Normalization (LayerNorm), which was originally introduced by Ba et al. to stabilize hidden feature distributions and accelerate optimization in deep neural networks. Since Vision Transformers rely extensively on LayerNorm before and after self-attention and feed-forward blocks, these normalization layers play an important role in maintaining robust feature representations under changing input distributions. More recently, efficient adaptation strategies have shown that selectively updating LayerNorm parameters can provide competitive performance while requiring only a very small fraction of the model parameters to be optimized. Ba et al. (2016); Zhao et al. (2024) demonstrated that tuning LayerNorm within transformer attention modules enables lightweight yet highly effective adaptation without the computational cost of full-network fine-tuning.

The LayerNorm configuration is also architecturally significant in this context: pre-LayerNorm designs have been shown to improve robustness and training stability compared to post-LayerNorm configurations (Kim et al., 2023), an observation that directly informs the adaptation strategy we propose.

Motivated by these findings, the present work adopts the established LayerNorm-only adaptation strategy as a computationally efficient adaptation mechanism during test-time inference. Rather than introducing a new adaptation strategy, this work investigates how existing lightweight TTA objectives behave across UAV datasets with varying semantic complexity under post-earthquake visual drift.

### 2.3 Robustness to visual degradation

There exists a substantial body of work on improving robustness to individual types of visual degradation. Motion-blur-specific architectural modifications have been developed for real-time UAV tracking, demonstrating that blur-aware inductive biases can preserve feature quality under platform instability (Wu et al., 2025). On the image restoration side, GAN-based dehazing networks employing global attention mechanisms and Laplacian-guided perceptual losses have achieved strong results for atmospheric haze removal in remote sensing imagery (Ali et al., 2025). These contributions are genuinely useful in their target settings.

The limitation that motivates our work is their narrowness: each such approach is designed around a single degradation type, typically requires retraining on degradation-specific data, and does not transfer to the multi-corruption setting of real post-earthquake operations. If a model was built to remove haze, it will not help when the dominant corruption is blur compounded by noise, and in field conditions, the dominant corruption changes not just across flights but within a single flight as the UAV moves through dust clouds and varying solar angles. A general adaptation mechanism that operates on the full corruption distribution, whatever form it takes, is needed.

### 2.4 Test-time adaptation

Early developments in test-time adaptation also explored perturbation-based robustness as a means of improving model generalization during inference without requiring access to target-domain labels. These studies demonstrated that prediction consistency under carefully designed perturbations can effectively mitigate the impact of distribution shift and laid the foundation for many subsequent TTA approaches (Sivaprasad and Fleuret, 2021).

TTA has developed into a principled and increasingly practical paradigm for handling distribution shift without any offline retraining. TENT, originally proposed by Wang et al., established the widely adopted entropy-minimization framework for fully test-time adaptation by updating normalization parameters using only unlabeled test data (Wang et al., 2021). Subsequent studies have extended this paradigm to remote sensing applications, including low-saturation confidence distribution-based adaptation for cross-domain aerial image classification (Liang et al., 2025). Complementary learning signals have been used to improve the precision of pseudo-label-based adaptation (Zeng et al., 2024), shift-aware class-specific thresholding has been proposed to handle non-uniform confidence profiles across classes (Karimi and Dibeklioglu, 2026), and consistency regularization across augmented views (as in MEMO-style methods) provides an alternative self-supervised signal that does not directly minimize entropy.

The effectiveness of this consistency-based paradigm was formally demonstrated through MEMO (Test-Time Robustness via Adaptation and Augmentation), which showed that multi-view augmentation combined with prediction consistency can substantially improve robustness under unseen distribution shifts without requiring retraining or target-domain supervision (Zhang et al., 2022).

Furthermore, Vision Transformer-specific adaptation strategies have recently received increasing attention. Kojima et al. proposed class-conditional feature alignment during test time to improve the robustness of Vision Transformers without retraining from scratch. This work was later extended through attention-based test-time adaptation, demonstrating that adapting transformer-specific attention representations during inference further improves robustness under distribution shift (Kojima et al., 2022, 2023).

Additionally, Sharpness-Aware Minimization (SAR) has emerged as a strategy to improve TTA reliability by flattening the loss landscape during inference, preventing the model from overfitting to noisy, drifted samples.

TTA has also found applications beyond standard classification, for instance in 6D pose tracking from video sequences (Tian et al., 2024), suggesting that the paradigm generalizes across different prediction structures.

Despite this activity, several gaps remain that are relevant to our problem. Evaluations have been concentrated on ImageNet-C and similar standard benchmarks; UAV disaster imagery has not been studied. Most existing studies evaluate individual adaptation methods in isolation, making it difficult to understand how their relative effectiveness changes with dataset characteristics such as semantic complexity and class cardinality.

Moreover, while methods like TENT, MEMO, and SAR offer distinct theoretical advantages, their comparative efficacy within specialized remote sensing tasks has not been established. Existing transformer-specific adaptation methods have primarily been evaluated on generic image recognition benchmarks, leaving their behavior under post-disaster UAV imagery and varying semantic complexity largely unexplored.

The choice between entropy minimization and consistency regularization has not been systematically compared as a function of classification-space dimensionality, even though this is a practically important design question when deploying TTA in systems with different numbers of output classes. Likewise, although lightweight LayerNorm-based adaptation has been explored in prior work, its empirical behavior across post-earthquake UAV imagery and datasets with substantially different semantic complexity has not been systematically investigated. The present work addresses these gaps through a unified cross-dataset evaluation of MEMO, TENT, and SAR under a common experimental protocol.

## 3 Methodology

### 3.1 Problem formulation

Let ${𝒟}_{s}={\left\{\left(x_{i},y_{i}\right)\right\}}_{i=1}^{N}$ denote a labeled source dataset of clean UAV images, where *x*_*i*_ is an input image and *y*_*i*_∈{1, …, *C*} its class label. A ViT model *f*_θ_, parameterised by θ, is trained on 𝒟_*s*_ to minimize the cross-entropy loss. At deployment, the model encounters an unlabeled test stream ${𝒟}_{t}={\left\{x_{j}\right\}}_{j=1}^{\infty }$ drawn from a shifted distribution *P*_*t*_(*x*)≠*P*_*s*_(*x*), induced by post-earthquake visual corruption. The goal of TTA is to identify a small parameter subset θ_*a*_⊂θ and update it using only the incoming unlabeled test samples, such that *f*_θ_ achieves improved accuracy on 𝒟_*t*_ without access to any ground-truth labels, stored target-domain data, or additional training rounds.

### 3.2 Framework overview

**Figure 2:**
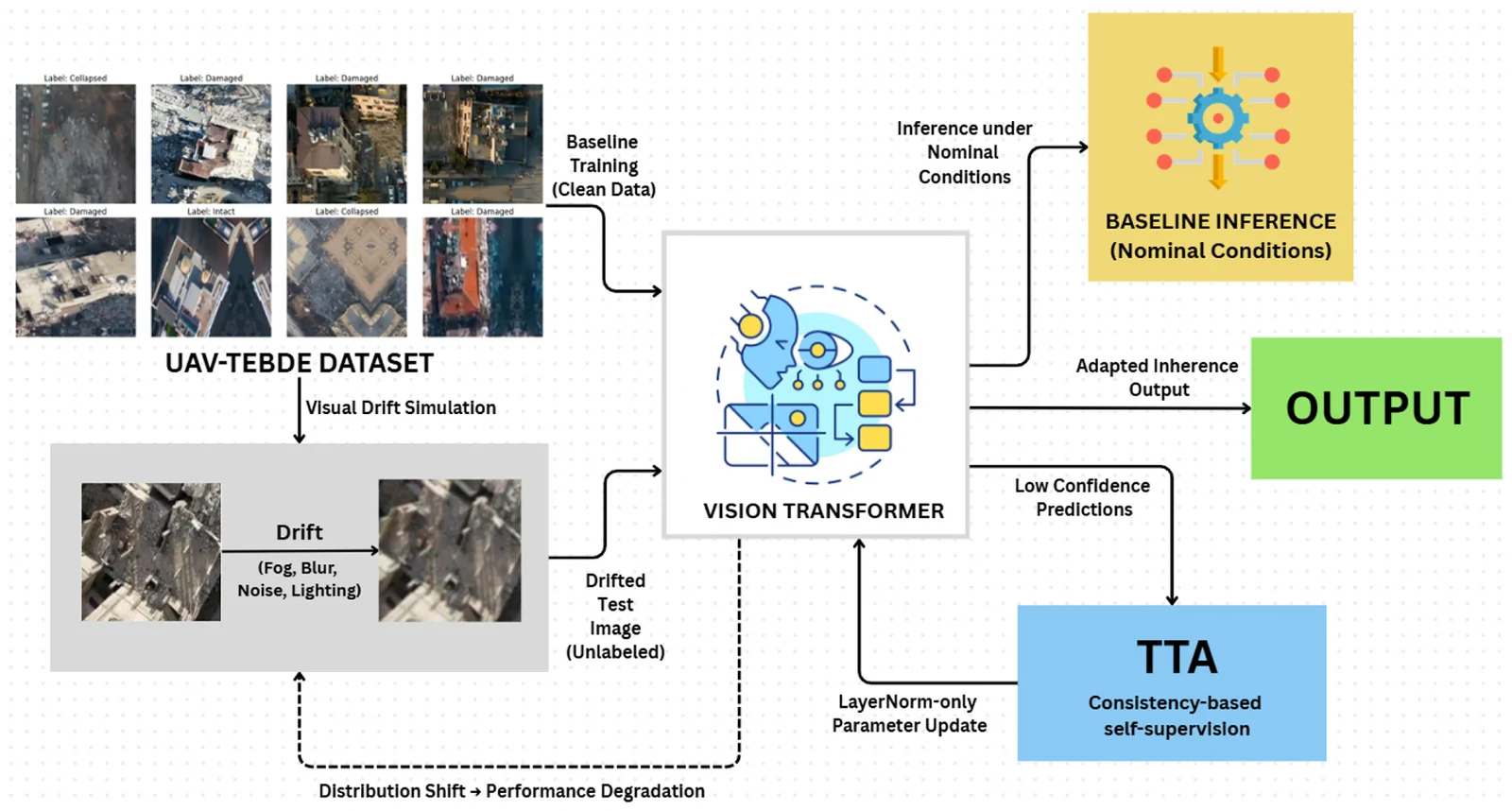
High-level three-phase pipeline: (1) offline training on clean UAV imagery, (2) nominal inference under post-earthquake visual drift, and (3) deployment-time test-time adaptation using lightweight LayerNorm updates. During inference, the selected TTA method (MEMO, TENT, or SAR) updates only the LayerNorm affine parameters, while all remaining Vision Transformer parameters remain frozen. Images were sourced from (Ref: Mowla, Md. Najmul (2025), “UAVs-based Turkey Earthquake Building Damage Estimation Dataset (UAVs-TEBDE)”, Mendeley Data, V1, doi: 10.17632/5m349hfvkb.1). The files associated with this dataset are licensed under a Creative Commons Attribution 4.0 International licence.

**Figure 3:**
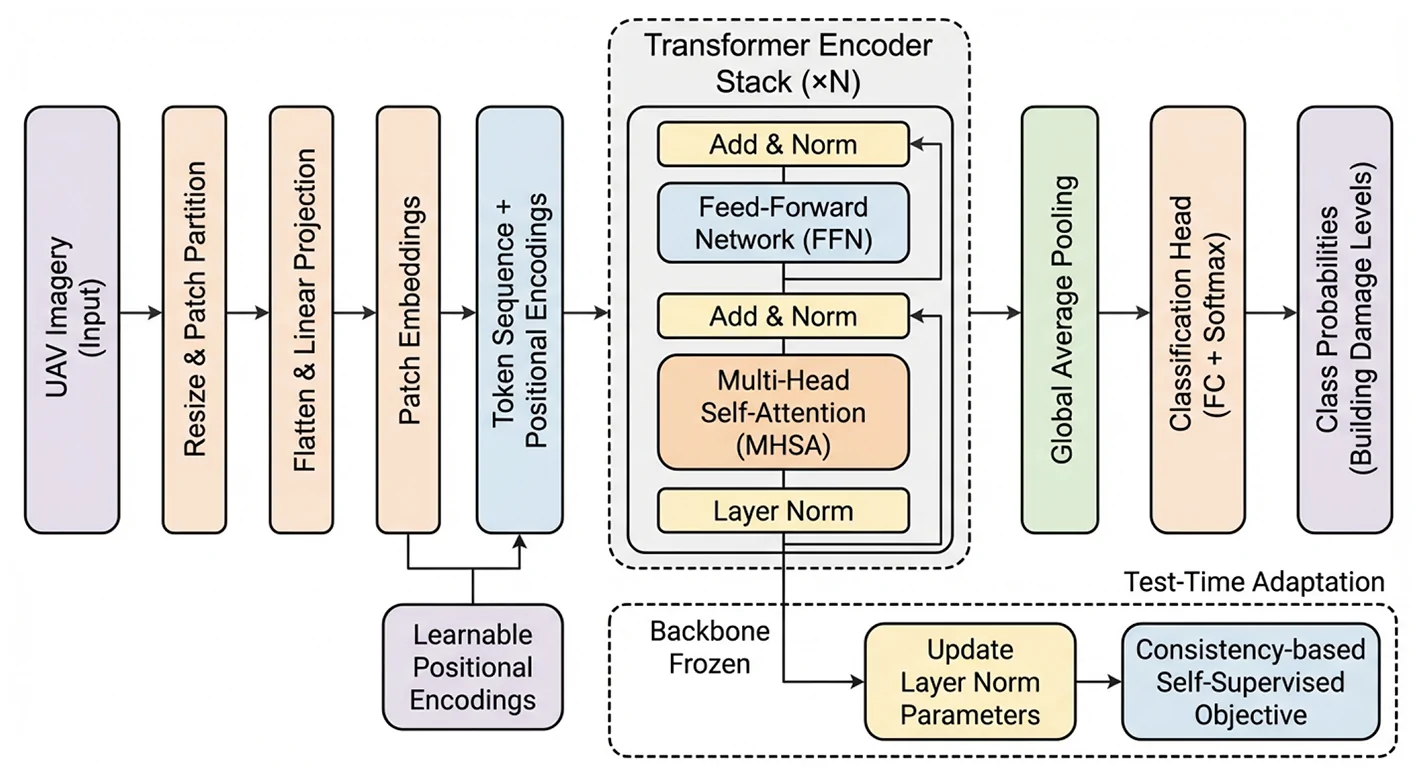
ViT-B/16 backbone with TTA stage. Patch embeddings are processed through stacked transformer encoder layers; LayerNorm parameters (highlighted) are the only components updated during inference. Depending on the selected TTA method, adaptation is performed using either consistency regularization (MEMO), entropy minimization (TENT), or sharpness-aware entropy minimization (SAR), while attention weights and feed-forward projections remain frozen.

The end-to-end pipeline is illustrated in Figure 2 and the ViT backbone together with the adaptation stage in Figure 3. The framework operates in three phases. In the first phase, a ViT-B/16 model is fine-tuned offline using clean labeled images from the source dataset 𝒟_*s*_. In the second phase, the trained model is deployed on unseen UAV imagery affected by post-earthquake visual drift, where performance degrades because the test distribution differs from the training distribution. In the third phase, test-time adaptation (TTA) is performed during inference without requiring target-domain labels.

Depending on the adaptation method under evaluation, the deployed model either minimizes prediction inconsistency across multiple augmented views (MEMO), minimizes prediction entropy (TENT), or performs sharpness-aware entropy minimization (SAR). Across all methods, only the LayerNorm affine parameters are updated during inference, while the transformer backbone, attention layers, feed-forward blocks, and classification head remain frozen. This common adaptation protocol enables a fair empirical comparison of lightweight TTA strategies under identical experimental conditions.

The ViT-B/16 backbone partitions each 224 × 224 input image into non-overlapping 16 × 16 patches and linearly projects each patch into a *d*-dimensional embedding. Learnable positional encodings are added to preserve spatial structure, and the resulting token sequence is processed through *L* transformer encoder layers. Each layer applies multi-head self-attention,


$$\begin{array}{l} \text{Attention}\left(Q,K,V\right)=\text{softmax}\left(\frac{QK^{⊤}}{\sqrt{d}}\right)V, \end{array}$$ (1)

followed by a position-wise feed-forward network, with LayerNorm and residual connections at each stage. The global self-attention mechanism allows every token to attend to all others, which is particularly valuable for aerial damage imagery where informative cues, collapse patterns, debris distribution, partial roof integrity, can be dispersed across large portions of the scene. Final token representations are aggregated via global average pooling and passed through a linear classification head with softmax to produce per-class probabilities.

### 3.3 Visual drift simulation

To evaluate the framework under controlled and reproducible conditions, we simulate post-earthquake visual drift at test time using four synthetic corruption types applied at severity levels *s*∈{0.1, 0.2, 0.3}: Gaussian noise with standard deviation σ = 0.25*s*, brightness and contrast perturbations proportional to *s*, Gaussian blur with kernel radius 5*s*, and synthetic fog with opacity 0.8*s*^2^. These four types were selected because they closely correspond to the dominant degradations actually observed in operational post-earthquake UAV flights: noise from thermally stressed sensors, illumination shifts from smoke and dust, optical blur from platform instability, and atmospheric haze from fires and debris clouds. The drift simulation used throughout this work applies static, spatially uniform, per-image corruptions to systematically isolate degradation variables. However, we acknowledge that real post-earthquake UAV footage involves temporally coherent, non-uniform degradations—smoke drifts across partial frames, sun angles shift dynamically, and platform vibrations vary non-linearly. Evaluating the framework against such dynamic sequences remains an important vector for full operational validation.

**Figure 4:**
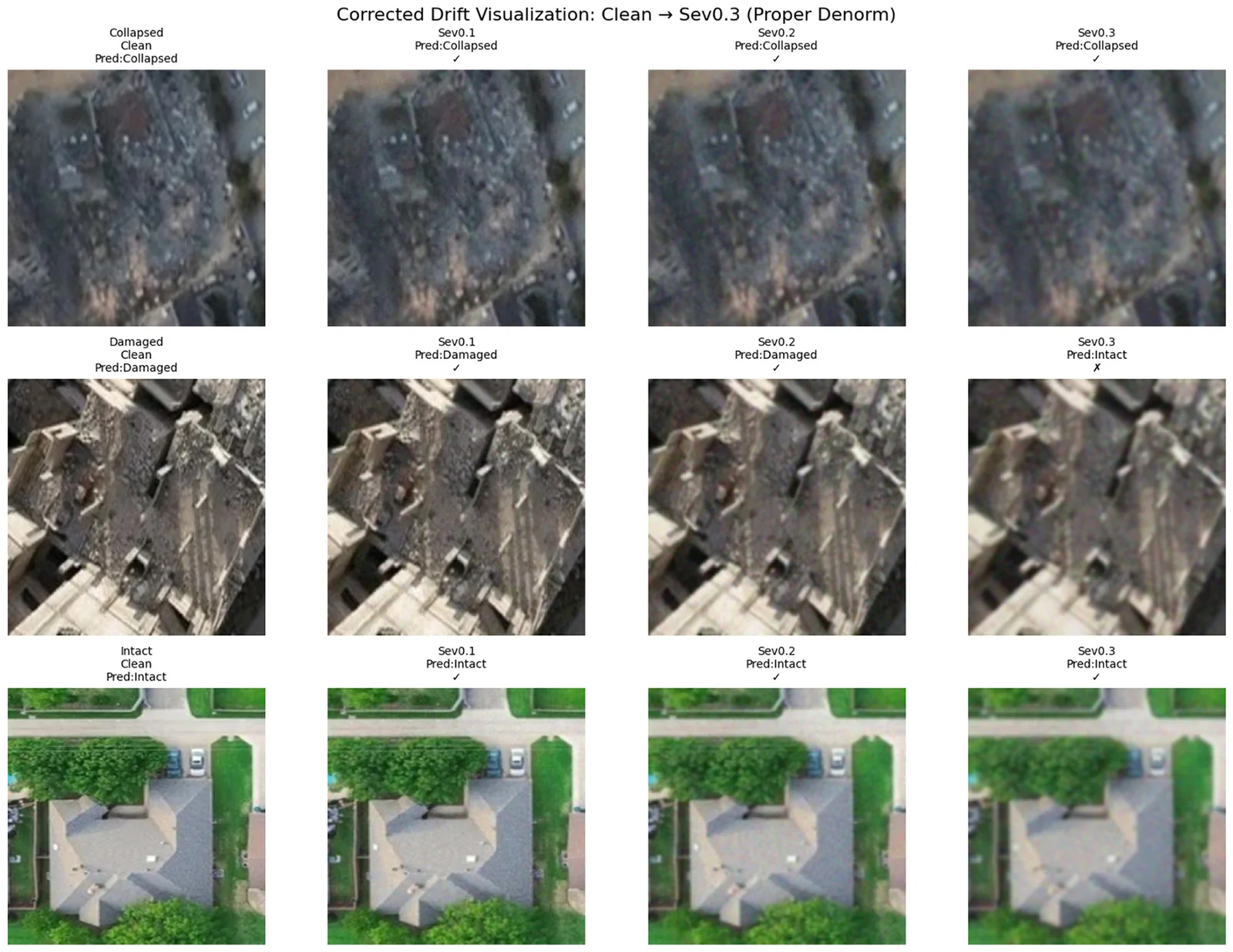
UAV-TEBDE drift progression from clean imagery (**left**) to severity 0.3 (**right**). Fine structural details are progressively lost, and the visual boundary between the Damaged and Collapsed categories is eroded, directly increasing confusion between these two classes. Images were sourced from (Ref: Mowla, Md. Najmul (2025), “UAVs-based Turkey Earthquake Building Damage Estimation Dataset (UAVs-TEBDE)”, Mendeley Data, V1, doi: 10.17632/5m349hfvkb.1). The files associated with this dataset are licensed under a Creative Commons Attribution 4.0 International licence.

No corrupted samples appear during training at any point; all drift is introduced exclusively at test time. This ensures that any accuracy drop in the drifted condition reflects genuine distributional shift rather than any mismatch in data augmentation strategy. Severity 0.3 is the primary experimental condition throughout this paper because it produces the most challenging deployment scenario and is most representative of real-world field conditions. The progressive visual effect of increasing severity across all three datasets is shown in Figure 4.

### 3.4 Consistency-based self-supervision

For the MEMO adaptation strategy, each incoming test image *x* is transformed into *K* stochastic augmented views ${\left\{x^{\left(k\right)}\right\}}_{k=1}^{K}$ using lightweight image perturbations. The objective is to encourage prediction consistency across these augmented observations, thereby improving robustness to distribution shifts without requiring target-domain supervision.

Each augmented view is independently passed through the current model to produce a predicted probability distribution $p^{\left(k\right)}=f_{\theta }\left(x^{\left(k\right)}\right)$. Because all views originate from the same underlying image, we expect their predictions to agree; any disagreement indicates that the model is responding to corruption-specific artifacts rather than the underlying semantic content. We therefore compute the mean prediction


$$\bar{p}=\frac{1}{K}\overset{K}{\underset{k=1}{\sum }}p^{\left(k\right)}$$

and minimize the consistency objective


$$\begin{array}{l} {ℒ}_{\text{cons}}=\frac{1}{K}\overset{K}{\underset{k=1}{\sum }}D_{\text{KL}}\left(p^{\left(k\right)}||\bar{p}\right). \end{array}$$ (2)

Minimizing ℒ_cons_ encourages stable predictions across multiple augmentations of the same test image, allowing the model to rely on corruption-invariant semantic features rather than degradation-specific artifacts introduced by visual drift.

### 3.5 Entropy minimization

Unlike MEMO, which enforces prediction consistency across multiple augmented views, the TENT adaptation strategy optimizes the model directly using prediction entropy. Distribution shift often causes the predicted class probabilities to become uncertain, particularly for datasets containing many visually similar classes. Entropy minimization counteracts this effect by encouraging confident predictions while adapting the model to the target distribution.

For a predicted class probability vector **p**, the entropy objective is defined as


$$\begin{array}{l} {ℒ}_{\text{ent}}=-\overset{C}{\underset{c=1}{\sum }}p_{c}\log p_{c}. \end{array}$$ (3)

Minimizing ℒ_ent_ encourages the model to produce low-entropy predictions on unlabeled target-domain samples while updating only the selected adaptation parameters.

In this work, the entropy objective is used exclusively by TENT, whereas the consistency objective described in the previous subsection is used exclusively by MEMO. The two adaptation strategies are evaluated independently under an identical experimental protocol in order to compare their effectiveness across datasets with different semantic complexity. No joint optimisation of the consistency and entropy objectives is performed.

### 3.6 LayerNorm-only parameter update

Consistent with recent lightweight test-time adaptation methods, adaptation is restricted to the affine parameters of the LayerNorm layers,


$$\begin{array}{l} \text{LN}\left(x\right)=\gamma \frac{x-\mu }{\sigma }+\beta ,\;\theta_{a}\leftarrow \theta_{a}-\eta \nabla_{\theta_{a}}ℒ, \end{array}$$ (4)

where γ and β denote the learnable scaling and shifting parameters, while μ and σ represent the feature mean and standard deviation computed during normalization.

Although several lightweight parameter subsets have been proposed for transformer adaptation, LayerNorm parameters were selected because they provide an effective trade-off between computational efficiency and adaptation capability. Furthermore, the ablation study presented in Experiments section demonstrates that LayerNorm-only adaptation consistently outperforms adapting only the classification head as well as jointly adapting the LayerNorm and classification head, while requiring updates to only a small fraction of the model parameters.

Updating only {γ, β} recalibrates the feature statistics throughout the transformer encoder while preserving the semantic representations learned by the attention layers and feed-forward networks. This substantially reduces the risk of catastrophic forgetting compared with adapting larger portions of the network.

For the ViT-B/16 backbone used in this work, LayerNorm-only adaptation updates approximately 38,400 trainable parameters, representing only a small fraction of the total model parameters and therefore introducing negligible computational overhead during inference.

Algorithm 1 details the complete inference procedure, including the per-image parameter reset that prevents drift accumulation across the test stream. This reset is an important design choice: without it, the LayerNorm parameters can drift progressively over a long test sequence, effectively overfitting to the accumulated history of test images rather than adapting freshly to each one. We recognize that this stateless reset trades off the ability to exploit temporal continuity across a drone's continuous flight path; however, it strictly guarantees stability against catastrophic forgetting during prolonged, unpredictable operations.

Algorithm 1 Deployment-Time Test-Time Adaptation for Drifted UAV Images 
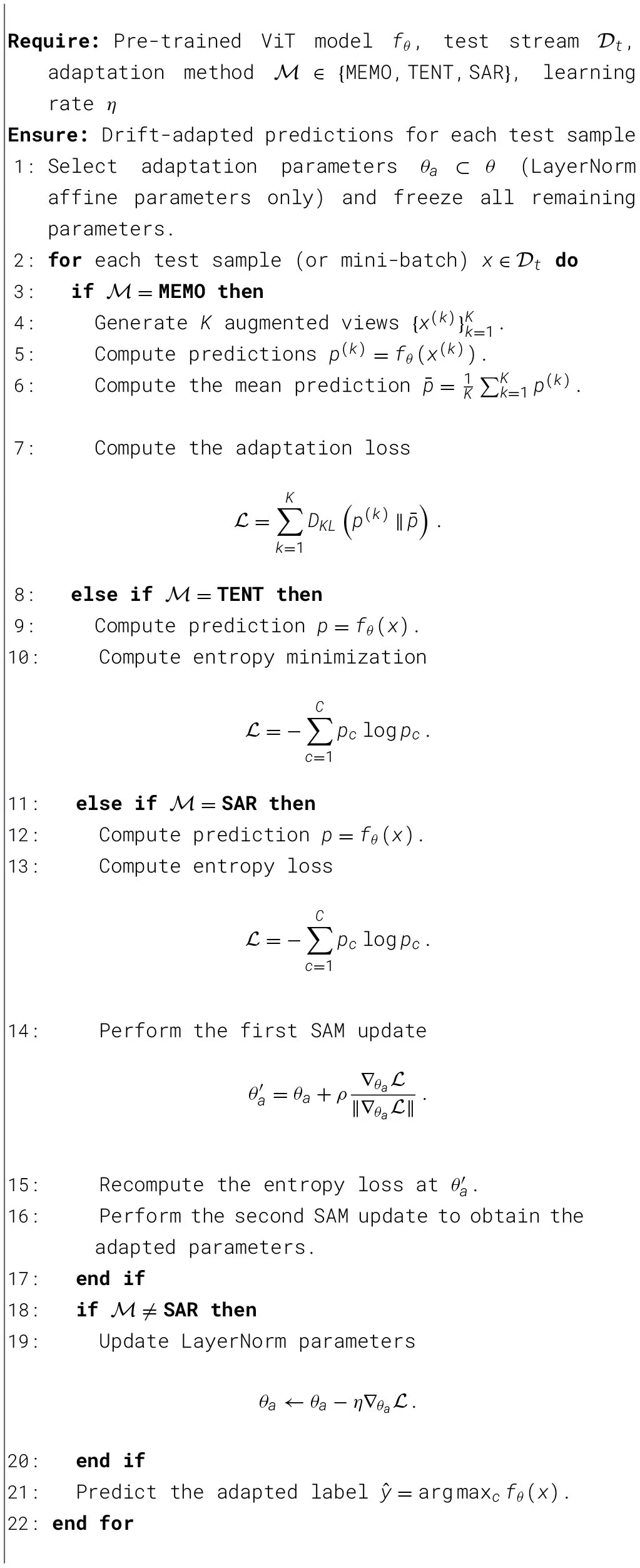

## 4 Experiments

### 4.1 Setup and dataset splits

We evaluate the proposed framework on three aerial imaging datasets that span meaningfully different classification complexities. UAV-TEBDE (Mowla, 2025) is a high-resolution post-earthquake building damage dataset collected in the aftermath of the 2023 Turkey earthquakes, covering three damage categories: Collapsed, Damaged, and Intact. It includes a large augmented training partition as well as a separate set of original UAV images held aside for deployment-style evaluation. AID (Xia et al., 2017) is a large-scale aerial scene classification benchmark spanning 30 scene categories with 10,000 images in total; it serves as the high-class-count stress test in our cross-dataset analysis. UC Merced (Yang and Newsam, 2010) provides 2,100 perfectly balanced land-use images across 21 classes, sitting between UAV-TEBDE and AID in terms of classification complexity. Full split details are provided in Table 1.

**Table 1:** Image counts per split across all three datasets.

Dataset	Split	Source	Images
UAV-TEBDE	Train	Augmented	13,200
	Validation	Augmented	1,650
	Test	Augmented	1,650
	Test	Original UAV	1,636
AID	Train	Original	8,000
	Validation	Original	1,000
	Test	Original	1,000
UC Merced	Train	Original	1,260
	Validation	Original	420
	Test	Original	420

The image size is fixed at 224 × 224 and normalized according to the statistics of ImageNet. The backbone model ViT-B/16 is initialized by ImageNet pretrained parameters and then trained on the training set of each respective dataset, using AdamW optimizer with cosine annealing learning rate schedule, with a learning rate of 3 × 10^−4^ for UAV-TEBDE (10 epochs), 1 × 10^−4^ for AID (15 epochs), and 1 × 10^−4^ for UC Merced (10 epochs). The drifted images do not participate in the training process in any way, but are only corrupted during testing.

### 4.2 Baseline performance

Before performing drift on our data sets, we must first show that there is sufficient capability in each model to accurately classify the data sets. The performance metrics for each model are shown in Table 2. In this case, all models obtain accuracy of more than 97%, with AID reaching a maximum of 98.90% when classifying all 30 classes. This will allow us to determine that there is sufficient capability in our ViT-B/16 backbone plus fine-tuned network. The full per-class F1 scores for UAV-TEBDE are given in Table 3. As expected, all three classes perform exceptionally well on this benchmark dataset. However, the Damaged category does show slightly lower F1 on the augmented subset when compared to the other two. This is not surprising since the ambiguous nature of moderate damage levels means that some instances may be visually indistinguishable from Collapsed and Intact examples. This is particularly true for buildings that have only partially collapsed.

**Table 2:** Baseline accuracy on clean test sets across all three datasets.

Dataset	Classes	Accuracy
UAV-TEBDE	3	0.9811
AID	30	0.9890
UC Merced	21	0.9714

**Table 3:** UAV-TEBDE baseline with per-class F1.

Split	Acc.	F1 (C)	F1 (D)	F1 (I)
Test (augmented)	0.9539	0.953	0.934	0.974
Test (original)	0.9811	0.975	0.979	0.989

F1 (C), F1 (D), F1 (I) denote collapsed, damaged, and intact respectively.

### 4.3 Impact of visual drift

Tables 4–6 present the classification performance of the classifiers that have been learned under various degrees of drift in each dataset. What emerges from the three different datasets is quite remarkable. As the drift severity increases to 0.1, the change in performance is marginal compared to the baseline performance obtained using the uncorrupted data since the features learned by the model are strong enough to withstand small distortions. As severity level increases to 0.2, there is a slight decline in performance, which indicates that the distortion is now affecting the ability of the model to generalize due to out-of-distribution samples. However, once the severity increases to 0.3, there is a drastic decrease in the accuracy for all the datasets. For UAV-TEBDE, the drop in accuracy is 23.7 percent; in AID, the reduction is 20.7 percent, while in UC Merced, there is a 21.1 percent decline. It is interesting to note that the degree of the drop in performance is almost uniform for all three datasets despite having different classes. Thus, this can be explained by the disruption of the feature space irrespective of the number of classes involved.

**Table 4:** UAV-TEBDE: accuracy under simulated visual drift across severity levels.

Drift severity	Accuracy	Drop
0.0 (clean)	0.9811	–
0.1	0.979	↓0.3%
0.2	0.947	↓3.5%
0.3	0.735	↓**23.7%**

The bold value is the drop achieved at the highest severity which we tested (0.3), and this would be the value used for inference of model performance in drift without adaptation.

**Table 5:** AID: accuracy under simulated visual drift across severity levels.

Drift severity	Accuracy	Drop
0.0 (clean)	0.9890	–
0.1	0.952	↓3.7%
0.2	0.887	↓10.3%
0.3	0.784	↓**20.7%**

The bold value is the drop achieved at the highest severity which we tested (0.3), and this would be the value used for inference of model performance in drift without adaptation.

**Table 6:** UC Merced: accuracy under simulated visual drift across severity levels.

Drift severity	Accuracy	Drop
0.0 (Clean)	0.9714	–
0.1	0.952	↓2.0%
0.2	0.855	↓11.9%
0.3	0.767	↓**21.1%**

The bold value is the drop achieved at the highest severity which we tested (0.3), and this would be the value used for inference of model performance in drift without adaptation.

### 4.4 Test-time adaptation results

Tables 7–9 report performance at drift severity 0.3 for each dataset. To better contextualize the performance of the proposed framework against contemporary adaptation strategies, we expanded our baselines on the primary UAV-TEBDE dataset to include Sharpness-Aware Minimization (SAR), a robust method designed to improve test-time reliability by flattening the loss landscape. Due to compute constraints, SAR evaluation was strictly isolated to this primary dataset. The results tell a clear story that differs by dataset complexity.

**Table 7:** UAV-TEBDE: TTA results at drift severity 0.3.

Method	Accuracy	Δ
Drifted baseline	0.7347	–
TENT	0.7467	+1.2%
SAR	0.8551	+12.0%
MEMO-TTA	**0.8918**	+15.7%

MEMO achieves the largest recovery in this 3-class setting, outperforming both TENT and the broader SAR baseline. The bold value shows the highest accuracy achieved for a particular dataset using different methods.

**Table 8:** AID: TTA results at drift severity 0.3.

Method	Accuracy	Δ
Drifted baseline	0.779	–
TENT	**0.8590**	+8.0%
MEMO-TTA	0.6820	−9.9%

TENT is superior in this 30-class setting; MEMO degrades performance. The bold value shows the highest accuracy achieved for a particular dataset using different methods.

**Table 9:** UC Merced: TTA results at drift severity 0.3.

Method	Accuracy	Δ
Drifted baseline	0.767	–
TENT	**0.8429**	+7.6%
MEMO-TTA	0.7048	−6.2%

TENT again outperforms MEMO in this 21-class setting. The bold value shows the highest accuracy achieved for a particular dataset using different methods.

On UAV-TEBDE, MEMO recovers 15.7 percentage points (73.47% → 89.18%), substantially narrowing the gap toward the clean-data ceiling of 98.11%. SAR also demonstrates strong adaptability on this dataset: at a moderate drift severity of 0.2, SAR slightly improves accuracy to 0.9548, and under severe drift (severity 0.3), it yields a 12.0 percentage point recovery (73.47% → 85.51%). Both SAR and MEMO substantially outperform the standard TENT baseline, which produces only a marginal 1.2-point gain. However, SAR still falls short of MEMO's recovery. The reason for MEMO's dominance here is straightforward: with only three output classes, any cross-view prediction disagreement carries a strong and unambiguous signal that the model is being distracted by corruption rather than responding to scene content. Enforcing consistency across augmented views directly suppresses that distraction and steers the model toward the corruption-invariant features that distinguish, say, a collapsed building from a structurally intact one.

However, the scenario is totally reversed for AID and UC Merced, where TENT yields improvements of 8.0 and 7.6, whereas MEMO causes drops of 9.9 and 6.2 points. It is interesting to note that the problem with MEMO at increased number of classes is quite insightful, whereby, for instance, misleading the classifier into a plausible yet wrong class due to the existence of a drift in a certain augmented view such as an airport terminal for a stadium can be exacerbated by consistency enforcement, since the classifier will only become increasingly confident in its wrong decision. Entropy minimization, however, circumvents this pitfall since it has no preference whatsoever for one class over another but rather seeks low entropy output irrespective of its source within the distribution. The implication is clear, whereby method selection for TTA should take classification space dimensionality into account.

The drifted baseline (0.779) originates from an independent experimental run performed during the TTA evaluation. The slightly different value reported in Table 5 (0.784) was obtained during the earlier drift characterization experiments. The difference (< 1%) results from separate experimental runs and does not affect the comparative conclusions.

### 4.5 Feature space and confusion matrix analysis

**Figure 5:**
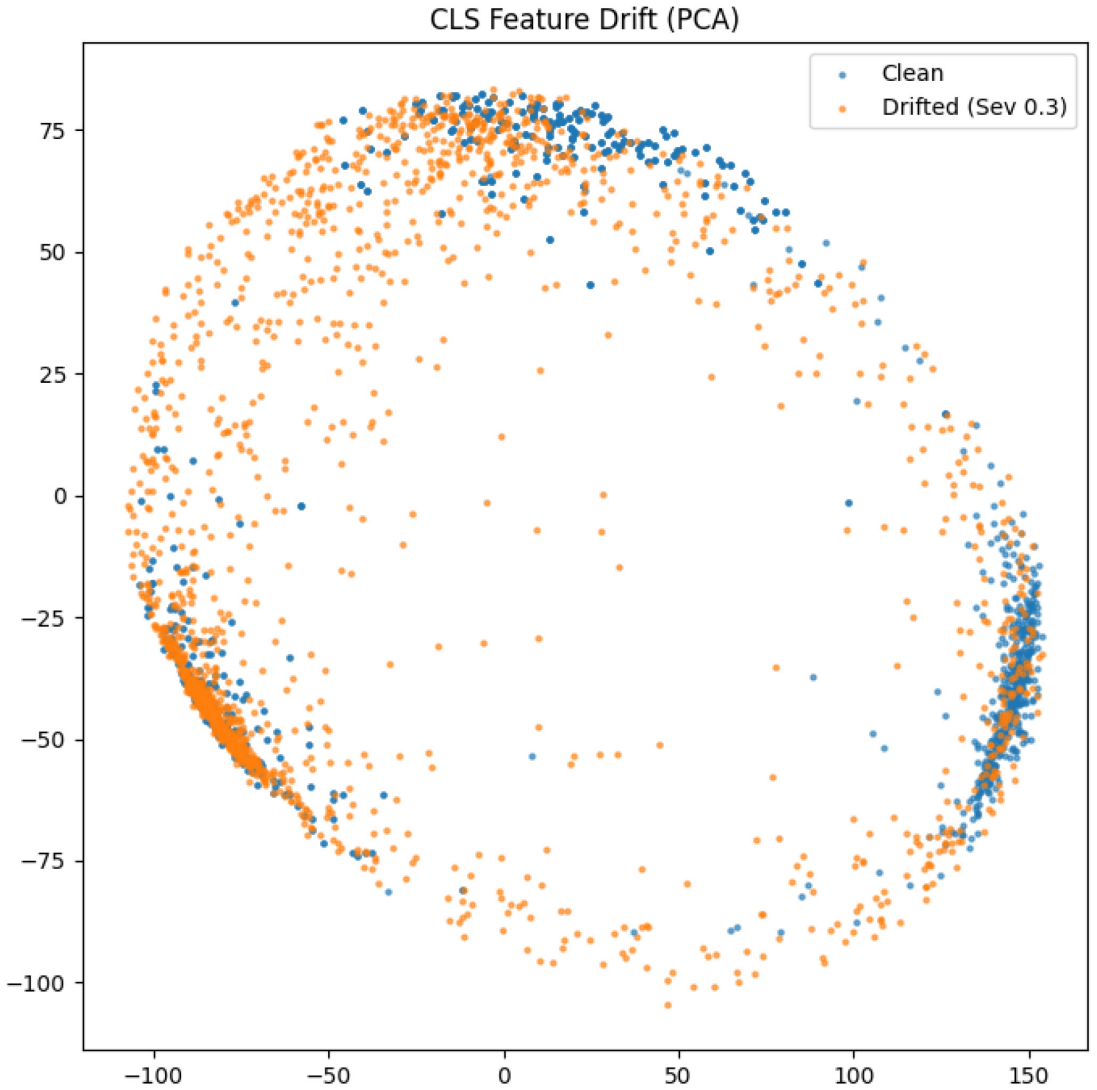
PCA of UAV-TEBDE final-layer token embeddings under clean, drifted (*s* = 0.3), and MEMO-adapted conditions. Clean clusters are compact and well-separated; drift disperses and merges them, especially at the Damaged–Collapsed boundary; adaptation partially restores the original geometry, confirming that LayerNorm updates recalibrate internal feature statistics.

To understand what is actually happening inside the model under drift, and why adaptation reverses it, we examine the internal token representations of the ViT under clean, drifted, and adapted conditions. PCA projections of token embeddings from the final layer for UAV-TEBDE examples are presented in Figure 5. For clean data, the three types of damage produce very clearly defined clusters in the 2D representation due to the discriminative nature of learned features. Under severe drift (severity 0.3), these clusters expand noticeably and begin to merge, particularly the Collapsed and Damaged categories, whose visual distinction depends on precisely the fine structural details that blur and haze destroy. The learned decision boundaries, calibrated to the clean-data geometry, no longer separate the shifted cluster positions, and accuracy falls accordingly.

After MEMO adaptation, the cluster structure partially recovers. The Collapsed and Damaged categories regain meaningful separation, and the overall geometry moves back toward the clean-data configuration, albeit not completely. Crucially, this is not a superficial logit correction, the change is visible in the penultimate token representations, well before the classification head is applied. This provides direct geometric evidence that LayerNorm parameter updates act by renormalising internal activations to fall within the statistical range the classification head expects, rather than simply adjusting the output layer to compensate for the shift.

**Figure 6:**
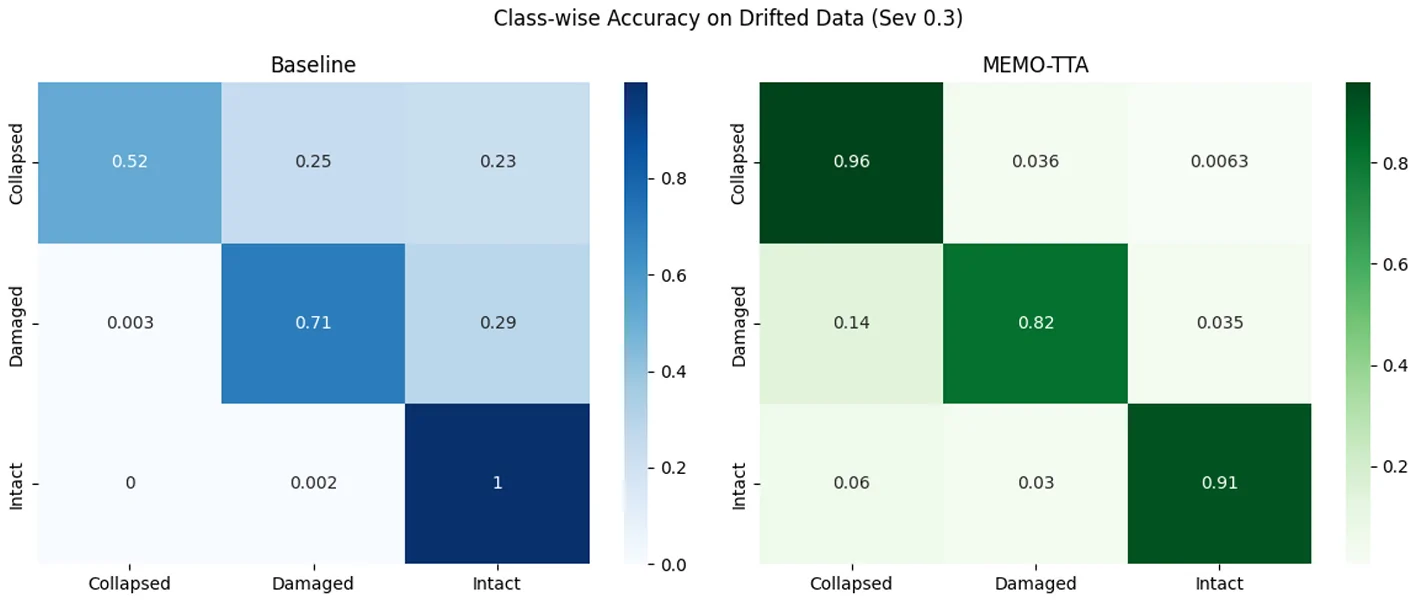
UAV-TEBDE confusion matrices under clean, drifted (*s* = 0.3), and MEMO-adapted conditions. Drift concentrates errors at the Damaged–Collapsed boundary, the most safety-critical class pair in post-earthquake assessment. Adaptation substantially reduces this confusion, with the adapted matrix closely approaching the clean-data structure.

Figure 6 presents the corresponding confusion matrices. The clean-condition matrix is nearly diagonal, strong and balanced performance across all three classes. Under drift, off-diagonal errors increase markedly. The most consequential of these errors is the confusion between Damaged and Collapsed: in an operational damage assessment setting, mislabelling a collapsed building as merely damaged can result in rescue teams being directed away from areas of highest need. This is precisely the failure mode that adaptation most effectively targets. After MEMO adaptation, the confusion matrix closely approaches the clean-data structure, with Damaged–Collapsed errors substantially reduced. The 15.7-point overall accuracy gain is therefore not spread uniformly across the confusion matrix; it is concentrated at exactly the class boundary that matters most for downstream rescue prioritization.

### 4.6 Cross-dataset summary

**Figure 7:**
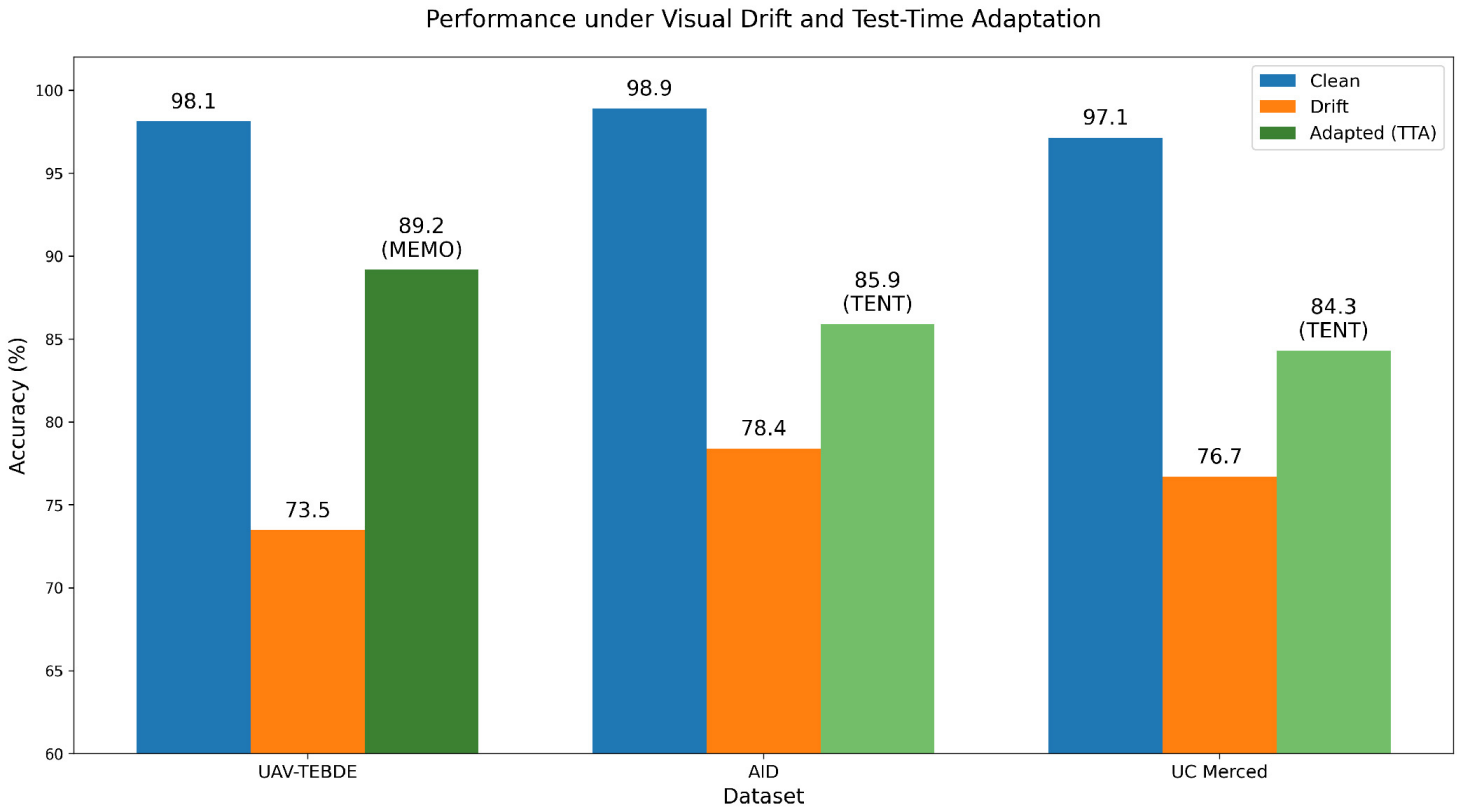
Cross-dataset accuracy under clean, drifted (*s* = 0.3), and best-adapted conditions for UAV-TEBDE, AID, and UC Merced. The consistent pattern of collapse under drift and recovery under adaptation demonstrates that lightweight LayerNorm-based TTA is an effective and general strategy across aerial imaging tasks of varying complexity.

Figure 7 summarizes accuracy across the clean, drifted, and best-adapted conditions for all three datasets. Three patterns hold consistently. All baseline models perform well above 97% on clean data. Severe drift pushes all three into the narrow 73–78% range regardless of dataset or class count, the universality of this collapse reinforces the point that the degradation mechanism operates at the level of feature geometry, not at the level of class-specific confusion. In all cases, the optimal TTA method is able to regain substantial lost accuracy, based on the relationship between the number of classes discussed earlier. The performance outcomes are presented in Table 10.

**Table 10:** End-to-end performance summary across all datasets.

Dataset	Clean	Drifted	Adapted	Method
UAV-TEBDE	98.11%	73.47%	**89.18%**	MEMO
AID	98.90%	78.40%	**85.90%**	TENT
UC Merced	97.14%	76.70%	**84.29%**	TENT

The best-performing TTA method is determined by classification-space size rather than any universal criterion. The adapted value is in bold to highlight the improved accuracy after drift.

### 4.7 LayerNorm parameter ablation

To justify the choice of adapting only the LayerNorm affine parameters during inference, an ablation study was performed on the primary UAV-TEBDE dataset under severe visual drift (severity 0.3). Since MEMO achieved the strongest adaptation performance on this dataset, the ablation was conducted within the MEMO framework while varying only the subset of trainable parameters. This isolates the effect of the adaptation parameters independently of the adaptation objective.

The results demonstrate that adapting only the LayerNorm parameters provides the most effective adaptation strategy. Updating only the classification head performs substantially worse than even the drifted baseline, indicating that modifying the output classifier alone cannot compensate for the feature distribution shift introduced by severe visual corruption. Jointly adapting the LayerNorm layers and the classification head also degrades performance relative to LayerNorm-only adaptation, suggesting that altering the classifier during unsupervised inference destabilizes the decision boundaries established during supervised training. These findings provide empirical justification for restricting adaptation to the LayerNorm affine parameters throughout the remainder of this study (Table 11).

**Table 11:** Ablation study of trainable parameter subsets for MEMO on UAV-TEBDE under drift severity 0.3.

Adaptation strategy	Trainable parameters	Accuracy
No adaptation	0	0.7347
Classification head only	2,307	0.5434
LayerNorm only	38,400	**0.8808**
LayerNorm + classification head	40,707	0.6119

The max accuracy achieved during the ablation study is in bold, which comes from “LayerNorm only”.

### 4.8 Repeated-run stability analysis

To evaluate the stability of the adaptation process, repeated inference experiments were conducted on the primary UAV-TEBDE dataset under drift severity 0.3. Since all three TTA methods operate directly on a fixed pretrained model without retraining, repeated evaluations quantify the stochasticity introduced by the adaptation procedure itself rather than variability arising from different model initializations. Owing to the computational expense of repeatedly performing test-time adaptation, this analysis was conducted on a randomly selected 25% subset of the UAV-TEBDE test set while preserving identical adaptation settings across all runs.

MEMO exhibits the lowest variability across repeated adaptation runs despite relying on stochastic data augmentations, indicating that its consistency-based optimization remains comparatively stable under repeated evaluation. TENT shows the largest variation, reflecting the greater sensitivity of entropy minimization to the confidence of individual predictions and the ordering of incoming test samples. SAR demonstrates intermediate stability by regularizing the optimization process through sharpness-aware updates. Although this experiment was performed on a representative subset rather than the complete test set for computational efficiency, the observed trends consistently support the robustness of the adaptation methods reported throughout the main experiments (Table 12).

**Table 12:** Repeated-run evaluation on a 25% subset of the UAV-TEBDE test set under drift severity 0.3.

Method	Mean accuracy	Std. Dev.
MEMO	**0.9112**	**0.0102**
SAR	0.8060	0.0232
TENT	0.7897	0.0342

Mean accuracy and standard deviation are reported over three independent adaptation runs. The max mean accuracy and min standard deviation is highlighted after the repeated run evaluation.

## 5 Discussion

Three findings from these experiments seem worth drawing out explicitly, as they have implications beyond the specific datasets and methods studied here.

First, clean-data benchmark scores are a poor predictor of deployment reliability in physically demanding operational settings. All three models in this study achieve well over 97% accuracy on their respective clean test sets, a result that would, in many evaluation contexts, be taken as a strong indicator of deployment readiness. Yet under the simultaneous multi-type corruptions of severe drift, each model loses more than 20 accuracy points. This is not random variation around a stable operating point; it is a systematic and reproducible collapse driven by geometric disruption of internal token representations, as the PCA visualizations make clear. Practitioners evaluating models for disaster assessment applications should not treat clean-benchmark performance as a proxy for operational robustness, and should explicitly test under realistic field conditions before deployment.

Second, our experiments indicate that LayerNorm-only adaptation provides an effective balance between computational efficiency and adaptation capability for the kind of shift considered here. The newly introduced ablation study further shows that LayerNorm-only adaptation outperforms both classification-head-only adaptation and joint LayerNorm-plus-head adaptation within the MEMO framework, providing empirical support for this design choice. Distribution shift from physical corruptions primarily manifests as a mismatch in activation statistics: the spatial attention patterns and the semantic content of feed-forward projections are not fundamentally wrong, but the scale and offset of activations at each layer no longer match the regime in which they were calibrated. Updating only γ, β corrects exactly this mismatch, layer by layer, without touching the semantic knowledge encoded in attention weights. The small number of parameters being updated (approximately 38,400 across the full ViT-B/16) also means the adaptation step is fast enough to be practically invisible in a real deployment pipeline. And the per-image reset ensures the model never accumulates drift across the test stream in a way that could compound errors over time, although this trades off the ability to exploit mission-level temporal continuity.

Third, the choice of adaptation objective is not a task-agnostic hyperparameter. The experimental results establish a clear and reproducible interaction: MEMO (consistency regularization) works substantially better than TENT in the three-class UAV-TEBDE setting, while TENT works substantially better, and MEMO actively hurts, in the 21- and 30-class settings. Understanding why helps clarify when each method is appropriate. In a small output space, cross-view prediction disagreement is a strong and specific signal; it means the model is genuinely uncertain between a small number of options, and enforcing consistency drives it toward the most stable one. In a large output space, consistent predictions do not carry the same guarantee: the model may be consistently and confidently wrong, because it has been reliably misled to a nearby but incorrect class. Entropy minimization does not have this failure mode. It exerts pressure toward any confident prediction, without reasoning about which class is predicted, and in a larger label space this is more likely to coincide with the correct class. This consistent behavior across three datasets suggests that the relative effectiveness of lightweight test-time adaptation methods depends on the semantic complexity of the classification task rather than there existing a universally superior adaptation strategy.

**Limitations**. The drift simulation used throughout this work applies static, spatially uniform, per-image corruptions. Real post-earthquake UAV footage involves temporally structured degradation, smoke drifts, sensor temperature changes over the course of a mission, sunlight angles shift, that a stateless per-image update cannot exploit or model. The framework's performance on such temporally coherent, non-uniform degradations remains an open and critical question for operational readiness. The evaluation is also restricted to image-level classification; tasks that are actually needed for operational disaster response, including instance segmentation, detection of individual structural failures, and spatial damage mapping, are not covered here. Finally, although the LayerNorm-only adaptation strategy proved effective in the present study and outperformed the alternative parameter subsets evaluated in our ablation experiments, there may be conditions of more extreme drift under which broader parameter groups or additional lightweight adaptation strategies would be required, potentially at the cost of increased forgetting risk.

## 6 Conclusion

We have presented a comprehensive empirical evaluation of lightweight label-free test-time adaptation strategies for Vision Transformers operating under post-earthquake visual drift. Using a common LayerNorm-based adaptation protocol, we systematically evaluated MEMO, TENT, and SAR across aerial imaging datasets of varying semantic complexity. On UAV-TEBDE, MEMO recovered 15.7 percentage points of accuracy under severe visual drift, improving performance from 73.47% to 89.18%, while the newly introduced LayerNorm parameter ablation and repeated-run experiments further validated the effectiveness and stability of the proposed evaluation protocol.

The cross-dataset analysis across UAV-TEBDE, AID, and UC Merced reveals something we think is practically important and not previously documented: the relative effectiveness of consistency-based vs. entropy-based adaptation depends systematically on the number of output classes. MEMO is the better choice for small class spaces; TENT is the better choice as class count grows. Treating TTA method selection as a task-agnostic decision, as it is often treated in the literature, may therefore lead to suboptimal choices in practice, highlighting the importance of considering dataset complexity when selecting lightweight adaptation strategies.

Looking ahead, the most compelling extension of this work is toward temporally-aware adaptation that exploits the sequential structure of a UAV video stream rather than treating each frame as an independent input. Flight sequences have natural temporal coherence that a per-image TTA framework currently ignores. Extensions to pixel-level tasks, semantic segmentation and instance-level damage detection, are also needed before the full operational loop from UAV deployment to automated rescue prioritization can be closed, alongside evaluating additional lightweight adaptation strategies for more challenging distribution shifts and expanding these adaptation principles to complex UAV-based 3D scene understanding tasks.

## 7 Limitations and future work

The results above show that LayerNorm-only adaptation meaningfully recovers accuracy under post-earthquake visual drift, but a few limitations are worth being upfront about before treating these numbers as field-ready.

The drift model itself is the first one. We simulate corruption through four synthetic, spatially uniform effects applied statically to each image, which gives us control and reproducibility but leaves open whether this actually matches real post-earthquake footage. In the field, degradation is rarely so tidy: smoke drifts across only part of a frame, sun angle shifts through a mission, and sensor noise creeps up as hardware heats during long flights. Testing the framework against real footage, ideally with some sense of how corruption actually varies in practice, is a needed next step. The framework's performance on such temporally coherent, non-uniform degradations remains an open and critical question for operational readiness. Related to this is our choice to reset LayerNorm parameters before every test image, which keeps drift from accumulating but also throws away the continuity a real flight provides, consecutive frames over the same site aren't independent, and a stateless reset ignores that. Future work should investigate temporally-aware adaptation strategies that preserve this continuity while maintaining adaptation stability. A short-memory variant, perhaps averaging parameters over a small recent window instead of resetting completely, could keep the stability benefit while still making use of that continuity, and is worth testing directly rather than assuming reset is the better choice.

We also only evaluate classification, while real disaster assessment usually needs pixel-level outputs, segmentation to map damaged areas, or detection to locate individual collapsed structures which are arguably more operationally relevant. Whether the LayerNorm-only adaptation strategy remains equally effective for these dense prediction tasks is genuinely unclear; dense prediction is more sensitive to feature-map shift than a single classification token, and our losses would need rethinking at the pixel or region level. Further out, the same idea, recalibrating a frozen backbone cheaply at inference time, could plausibly extend to large-scale 3D scene reconstruction from multi-view UAV imagery, where distributed reconstruction pipelines run into their own cross-flight drift. Specifically, investigating whether the proposed mechanisms can be used in complex UAV-based 3D scene understanding tasks like collaborative distributed learning for 3D Surface Gaussian Splatting and knowledge distillation-based distributed dynamic 3D Gaussian Splatting remains a promising future direction.

Although the present work now includes an ablation comparing LayerNorm-only adaptation against classification-head-based alternatives, additional lightweight adaptation strategies remain largely unexplored. Future work should investigate whether other parameter subsets, such as attention biases, depth-specific normalization layers, or lightweight adapter modules, provide improved robustness under more severe distribution shifts while preserving the computational efficiency required for deployment.

Finally, our baselines are MEMO and TENT, which served the comparison we wanted to make but leave out test-time batch normalization, adapter-based tuning, robust pretraining, restoration preprocessing, and other remote-sensing-specific TTA methods. A more comprehensive comparison with these contemporary adaptation methods would better contextualize the relative strengths of lightweight TTA approaches for remote sensing applications. Beyond the parameter subsets evaluated in this work, future studies should also investigate additional lightweight adaptation mechanisms and broader empirical comparisons across diverse remote sensing benchmarks. And we haven't measured what any of this costs on actual UAV hardware: per-frame latency, memory, energy consumption, power draw, or stability over a multi-hour mission. That last point matters more once a single UAV becomes a coordinated fleet, where secure data handling and power budgets start to matter as much as accuracy.

## Data Availability

The original contributions presented in the study are included in the article/supplementary material, further inquiries can be directed to the corresponding author.
